# Oral corticosteroid prescribing habits of Canadian Otolaryngologist-Head and Neck Surgeons

**DOI:** 10.1186/s40463-016-0128-4

**Published:** 2016-02-29

**Authors:** Saad Ansari, Brian W. Rotenberg, Leigh J. Sowerby

**Affiliations:** Department of Otolaryngology-Head & Neck Surgery, Western University, London, ON Canada; Department of Otolaryngology-Head & Neck Surgery, St. Joseph’s Hospital, Room B2-501, 268 Grosvenor Street, London, ON N6A 4V2 Canada

**Keywords:** Oral corticosteroids, Otolaryngology, Dosing regimen, Evidence-based medicine, Chronic Rhinosinusitis, Taper, Sudden Sensori-neural Hearing Loss, Facial Nerve Palsy

## Abstract

**Background:**

Oral corticosteroids (OCSs) are widely prescribed in Otolaryngology-Head & Neck surgery (OtoHNS). There is evidence in the literature regarding specific dosing regimens. However, it is not known to what extent these recommendations are being implemented in practice.

**Methods:**

An anonymous online survey was sent to Canadian Society of Otolaryngology-Head and Neck Surgery members (*N* = 696). Dosing, frequency and tapering of OCSs were assessed in acute rhino-sinusitis (ARS), chronic rhino-sinusitis with (CRSwP) and without polyps (CRSsP), sudden sensori-neural hearing loss (SSNHL), and idiopathic facial nerve/Bell’s palsy (IFN). Participants were asked to complete for conditions treated and results were compared with current guidelines. Development of prescribing habits and observed complications were also explored.

**Results:**

124 surveys (18 %) were completed. In CRSwP (*N* = 98), the median dose was 50 mg (Range: 10–100 mg) and the average duration was 8 days (Range: 1–21 days). In CRSsP (*N* = 29), the median dose was 50 mg (Range: 20-80 mg) and the average duration was 8 days (Range: 1–14 days). In SSNHL (*N* = 118), the median dose was 60 mg (Range: 10–120 mg) and the average duration was 10 days (Range: 1–21 days). In IFN (*N* = 108), the median dose was 50 mg (Range: 10–100 mg) and the average duration was 10 days (Range: 1–21 days). Tapering dosages were used in treating CRSwP (64 %), CRSsP (62 %), ARS (44 %), SSNHL (60 %) and IFN (53 %). Respondents most frequently perceived “Mentor/Preceptor Guidance” as a source of their prescribing habits.

**Conclusion:**

There is significant heterogeneity in OCS prescribing habits despite the availability of fairly consistent evidence in the literature for some of the surveyed conditions. Improvements in standardization should be made with the aim of enhancing outcomes and reducing complications.

## Background

Oral corticosteroids (OCSs) are commonly used for a variety of different diseases. In 2013, Overman et al. determined that 1.2 % of the US population over age 20 were prescribed OCSs, translating into over 2.5 million people [1]. In the field of Otolaryngology-Head & Neck Surgery (OtoHNS), OCSs are used for several indications, including chronic rhinosinusitis, sudden sensori-neural hearing loss, and idiopathic facial nerve, or Bell’s, palsy.

Due to the significant side effect profile, OCSs are often reserved as part of maximal medical therapy when other treatments have not been successful. Complications such as avascular necrosis of the hip, immunodeficiency, weight gain, insomnia, and psychosis have been well described in the literature [2]. While some are idiosyncratic reactions, most side effects have been shown to correlate with increasing doses of OCSs [3]. Therefore, the dosing of OCSs is important to provide maximal benefit while minimizing potential side effects.

A 2013 UK study by Sylvester et al. qualitatively characterized OCS prescribing habits as part of maximal medical therapy in chronic rhinosinusitis, with 66 % rarely or never prescribing OCSs [4]. Of those that did, 42 % utilized a duration of 0-5 days, 29 % for 6–10 days and 29 % for 11–15 days - suggesting that there is significant heterogeneity in prescribing practice in the UK. Kaszuba & Stewart showed that 36 % of Otolaryngologist-Head & Neck surgeons used OCSs in chronic rhinosinusitis [5]. This study also concluded that maximal medical management was influenced mainly by personal clinical experiences rather than evidence in the literature. Similar studies have not been conducted in Canada regarding OCS prescribing habits.

The aim of this study is to characterize OCS prescribing habits of Canadian Otolaryngologist-Head & Neck surgeons for chronic rhinosinusitis, acute rhinosinusitis, sudden sensori-neural hearing loss, and idiopathic facial nerve (Bell’s) palsy. While the evidence for dosage is heterogeneous for some conditions, such as chronic rhinosinusitis, a much clearer consensus on dosage exists for others. This study hopes to provide a glimpse into the status of evidence-based practice for prescribing OCSs in Canadian OtoHNS.

## Methods

Formal ethics approval was obtained through the Western University Research Ethics Board (Board number 105523) prior to beginning the study. An anonymous nationwide survey was conducted through an online survey program (QuestionPro.com®) and electronically distributed to all active members of the Canadian Society of Otolaryngology – Head and Neck Surgery’s mailing list (*n* = 696) between October and November 2014. A reminder email was sent approximately 3 months after initial distribution and participants were incentivized with a gift card draw at completion of the study. A cover letter accompanied the survey to outline issues of consent and to disclose the study’s goal of characterizing prescribing habits of Canadian Otolaryngologist-Head & Neck surgeons. Respondents were notified that no identifying data would be included in the study.

**Fig. 1:**
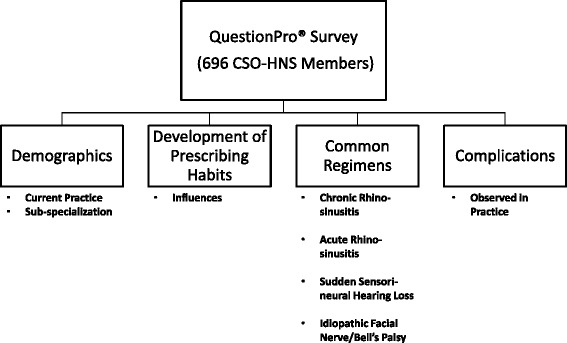
Components of the online survey. Respondents were asked to answer questions regarding personal demographics, development of prescribing habits, and specific dosing regimens for CRSwP, CRSsP, ARS, SSNHL, and IFN. Respondents were also asked to describe complications observed in their practice

Demographic data collected focused on the nature of respondents’ current practices. Indication, initial dose, duration, frequency, and use of taper were described for five common indications of OCSs in OtoHNS. These were chronic rhinosinusitis with polyposis (CRSwP), chronic rhinosinusitis without polyposis (CRSsP), acute rhinosinusitis (ARS), sudden sensori-neural hearing loss (SSNHL), and idiopathic facial nerve (Bell’s) palsy (IFN). Respondents were also asked to describe influences that helped to establish personal dosing regimens as well as observed complications with the use of OCSs in practice (Fig. 1).

## Results

Out of the 696 survey requests sent to members of the Canadian Society of Otolaryngology-Head and Neck Surgery, 124 surveys were returned fully completed (18 % response rate). The majority of respondents were surgeons in active practice (87 %). There were slightly more community practitioners (55 %) than academic practitioners (40 %). The remaining 5 % had a mixed academic and community practice. The two most-represented subspecialties were General Otolaryngology (52 %) and Rhinology (27 %). These were followed by Pediatrics (23 %), Head and Neck (20 %), Otology (19 %), Facial Plastic and Reconstructive Surgery (17 %), Laryngology (12 %) and other (4 %). The average number of OCSs prescribed by respondents was 6 prescriptions per month (range: 0–40 prescriptions).

**Fig. 2:**
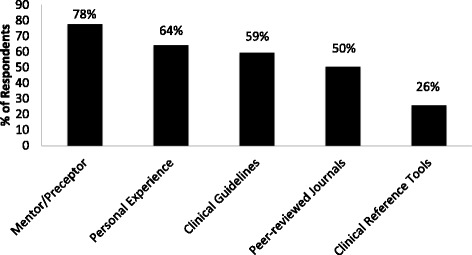
Influences for current oral corticosteroid prescribing habits of survey respondents

The most common self-described OCS prescribing influence was a respondent’s mentor or preceptor (78 %), followed by personal experience (64 %) and clinical guidelines (59 %) (see Fig. 2).

### Chronic rhinosinusitis

**Fig. 3:**
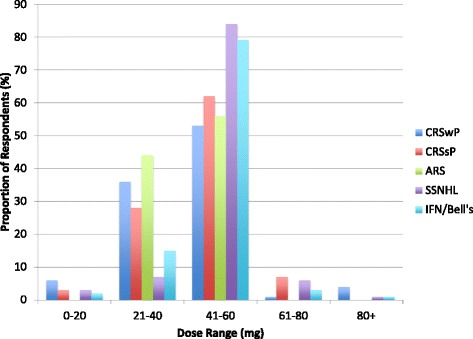
Initial doses described by respondents for all conditions. Chronic Rhinosinusitis with Polyposis (*Blue*); Chronic Rhinosinusitis without Polyposis (*Red*); Acute Rhinosinusitis (*Green*); Sudden Sensori-neural Hearing Loss (*Purple*); Idiopathic Facial Nerve (Bell’s) Palsy (*Light Blue*)

**Fig. 4:**
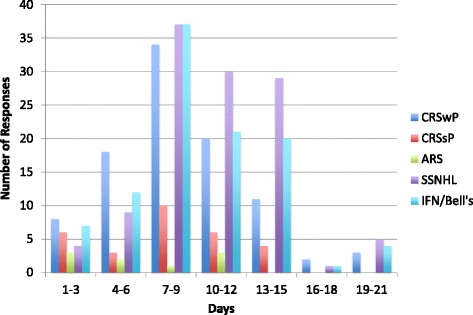
Durations described by respondents for all conditions. Chronic Rhinosinusitis with Polyposis (*Dark Blue*); Chronic Rhinosinusitis without Polyposis (*Red*); Acute Rhinosinusitis (*Green*); Sudden Sensori-neural Hearing Loss (*Purple*); Idiopathic Facial Nerve (Bell’s) Palsy (*Light Blue*)

In chronic rhinosinusitis, 79 % of respondents prescribed OCSs for CRSwP and 23 % for CRSsP. The most common reasons in both cases were “as part of maximal medical therapy” and “symptomatic exacerbation”. In CRSwP, there were 12 unique doses described by respondents. The median starting dose was 50 mg with a range between 10 and 100 mg. The average duration was 8 days with a range between 1 and 21 days. In CRSsP, there were 7 unique doses used by respondents. The median dose was 50 mg with a range between 20 and 80 mg. The average duration was 8 days with a range between 1 and 14 days. Tapers were used by approximately two-thirds of those that used corticosteroids for both conditions (Table 1, Figs. 3 & 4).

**Table 1:** Dosing regimens responses for CRS, ARS, SSNHL, and IFN/Bell’s Palsy. This table shows the use of OCSs, number of unique dosing regimens reported, median starting dose, average duration, and taper utilization for CRSwP, CRSsP, ARS, SSNHL, and IFN

Indication	Use (% of respondents)	Unique dosing regimens	Median dose with ranges	Duration with ranges	Taper (% of respondents)
CRSwP	79	12	50 (10–100)	8 (1–21)	64
CRSsP	23	7	50 (20–80)	8 (1–14)	62
ARS	7	9	50 (25–60)	6 (2–10)	44
SSNHL	95	11	55 (10–120)	10 (1–21)	60
IFN/Bell’s Palsy	87	9	50 (10–100)	9 (1–21)	53

### Acute rhinosinusitis

In acute rhinosinusitis, 7 % of respondents reported prescribing OCSs. All nine respondents had unique dosing regimens. The median dose was 50 mg with a range between 25 and 60 mg. The average duration was 6 days with a range between 2 and 10 days. A total of 44 % used a taper (Table 1, Figs. 3 & 4).

### Sudden sensori-neural hearing loss

In SSNHL, an overwhelming majority of respondents used OCSs (95 %). The most common reason to initiate therapy was to improve hearing. There were 11 unique dosing regimens described. The median starting dose was 55 mg with a range between 10 and 100 mg. The most common starting dose was between 40 and 60 mg of prednisone, with 98 (80 %) of respondents doing so. The average duration was 10 days with a range between 1 and 21 days. In this group of respondents, 60 % utilized a taper (Table 1, Figs. 3 & 4). Approximately 80 % (98/123) of respondents used a starting dose between 40 and 60 mg.

### Idiopathic facial nerve (Bell’s) palsy

For IFN palsy, 87 % of respondents prescribed OCSs. There were 9 unique dosing regimens described. The median dose was 50 mg with a range between 10 and 100 mg. Only 83 (67 %) of respondents used a starting dose of prednisone between 40 and 60 mg. The average duration was 9 days with a range between 1 and 21 days. Of these respondents, 53 % employed a taper (Table 1, Figs. 3 & 4).

### Complications

**Fig 5:**
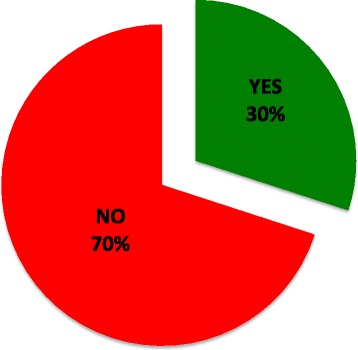
Percentage of respondents who have observed a complication with oral corticosteroids in their practice. Thirty percent of respondents answered “Yes” (*Green*) and 70 % answered “No” (*Red*)

**Fig. 6:**
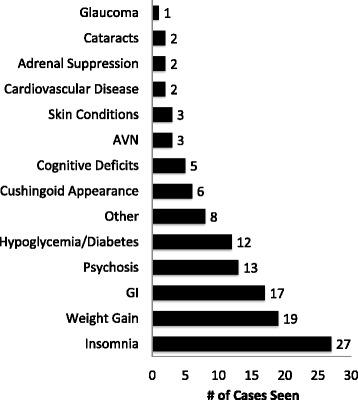
Complications observed with the use of oral corticosteroids by respondents

When asked about complications observed in practice, 30 % of respondents had personally managed patients with complications from the use of OCSs (Fig. 5). Insomnia, weight gain and gastrointestinal symptoms were most commonly described. Other rare complications were also mentioned including avascular necrosis, adrenal suppression, and hypoglycaemia (Fig. 6).

## Discussion

This study has characterized the corticosteroid prescribing habits of Canadian Otolaryngologist-Head & Neck surgeons for five common conditions in OtoHNS. The 18 % response rate, which is similar to previous survey studies of Canadian Otolaryngologist-Head and Neck surgeons, provides a well-balanced representation of community and academic practice respondents [6–9]. The results demonstrate the significant variability in prescribing habits, as evidenced by the high number of unique dosing regimens for each condition. Even greater variation would likely have been seen with a higher response rate, as the findings of this study are in keeping with the heterogeneity seen in previous surveys for OCS usage [4]. This is likely to be expected as mentor and preceptor habits along with personal experience were most commonly selected as being influences on respondents’ prescribing habits.

For CRSwP, the 2011 Canadian Society of Otolaryngology-Head and Neck Surgery (CSO-HNS) clinical practice guidelines suggest a two-week course of OCS to aid in treatment [10]. It does not elaborate on dosing regimens. A 2013 International Forum of Allergy & Rhinology review by Poetker et al. provides a more specific regimen of 25–60 mg for 7–14 days with Level A evidence from Level 2–4 studies [11]. The Canadian Family Physician guidelines also provide a dosing regimen of a two-week course of prednisone with a taper [12]. An example regimen was given for 30 mg per day for 4 days, then reduce the dose by 5 mg every 2 days for 10 days. With these guideline recommendations in mind, it is not surprising that this study noted 12 unique regimens and a very wide range of doses (10–100 mg). The guidelines were unable to commit to a specific regimen, as there is significant heterogeneity in the literature. For CRSsP, the same 2011 CSO-HNS guidelines do not provide any dosing or duration suggestions but provide a statement supporting the use of a short course of OCSs in this condition [10]. The 2013 IFAR review by Poetker et al. provide an optional level C recommendation suggesting 40–60 mg for 10–14 days [11]. This heterogeneity and weakness in the literature for CRSsP, as well as CRSwP, may be the reason for wide variety of prescribing habits observed in this study. Regardless, some of the dosing regimens described by respondents fall outside of the broad spectrum of recommendations - either not providing patients with benefit if too low, or exposing them to unnecessary potential risk if too high.

The dosing and duration recommendations of corticosteroid for SSNHL and IFN palsy, on the other hand, is more granular. For SSNHL, the American Academy of Otolaryngology–Head and Neck Surgery clinical guidelines recommend the use of OCSs at 60 mg for 10–14 days from level B evidence [13]. In IFN palsy, the Canadian Medical Association Journal guidelines provide a strong recommendation for a five-day course of 60 mg per day followed by a five day taper, reducing the previous day’s dose by 10 mg per day [14]. It is also suggested that a total dose of over 450 mg is necessary to obtain optimal benefit. Only 37 % of respondents prescribed a dose over this total amount. The evidence supporting these recommendations is stronger than the literature for CRS, and should provide prescribers with greater guidance as to the ideal dose and duration of corticosteroid. The results of this study, in spite of stronger guideline recommendations, demonstrate that there still is a range in doses and duration for both conditions with only 80 and 67 % of respondents using the recommended initial dose in SSNHL and IFN respectively. Prescribing below the guideline recommendations could potentially not provide patients with the intended benefit, while prescribing above could be exposing patients to unnecessary risk without any further additional benefit.

Evidence for corticosteroid use in acute rhinosinusitis is against routine use and not supported by current guideline recommendations [10, 15]. In spite of this, 7 % of respondents in this survey used OCSs in their treatment regimen, likely exposing patients to unnecessary risk without benefit.

Overall, this study was able to characterize current in-practice dosing regimens for five common conditions in which OCSs are used in OtoHNS. Further study needs to be performed to determine the optimal dose and duration for OCSs in CRS. However, there is significant heterogeneity in OCS prescribing habits irrespective of the strength of guideline recommendations regarding dose and duration. Perhaps greater emphasis is needed to encourage adherence to evidence-based practice to optimize medical therapy for patients.

Although this study aids in characterizing the dosing regimens of OCSs used in OtoHNS in Canada, it is self-reported. Hawthorne effect bias is likely present when respondents were completing this survey. Therefore, the results may be an under-estimation of the variability in the prescribing habits. This would further strengthen the conclusions made from this study. Additionally, it was difficult to parse out the reason for respondents to select “Yes” or “No” when replying to questions regarding the use of OCSs in each of the conditions mentioned in the survey. Some may have interpreted it as a question inquiring about the use of OCSs in their practice while others may have understood it as a question of whether they believed in prescribing OCSs for each specific condition. This would have potentially skewed the results of this question for each of the conditions. Further clarification of the question would have been optimal along with a third option of “Do not prescribe in my practice”.

## Conclusion

OCSs are widely used in OtoHNS. This study provides a glimpse into the in-practice prescription habits of Canadian Otolaryngologist-Head & Neck surgeons for chronic rhinosinusitis with and without polyps, acute rhinosinusitis, sudden sensori-neural hearing loss, and idiopathic facial nerve palsy. As expected, there is a wide range of dosing regimens currently being used – some being within the recommended guidelines and others that are not. Further research to standardize prescribing habits in order to optimize patient outcomes and minimize potential risk from unnecessarily high doses of corticosteroid would be beneficial.

## Abbreviations

ARS: acute rhinosinusitis
CRSsP: chronic rhinosinusitis without polyposis
CRSwP: chronic rhinosinusitis with polyposis
IFN: idiopathic facial nerve (Bell’s) Palsy
OCS: oral corticosteroid
OtoHNS: Otolaryngology-Head and Neck Surgery
SSNHL: sudden sensori-neural hearing loss
